# Development and Validation of a Prognostic Model for Cognitive Impairment in Parkinson’s Disease With REM Sleep Behavior Disorder

**DOI:** 10.3389/fnagi.2021.703158

**Published:** 2021-07-12

**Authors:** Fangzheng Chen, Yuanyuan Li, Guanyu Ye, Liche Zhou, Xiaolan Bian, Jun Liu

**Affiliations:** ^1^Department of Neurology and Institute of Neurology, Ruijin Hospital, Shanghai Jiao Tong University School of Medicine, Shanghai, China; ^2^Department of Pharmacy, Ruijin Hospital, Shanghai Jiao Tong University School of Medicine, Shanghai, China; ^3^CAS Center for Excellence in Brain Science and Intelligence Technology, Ruijin Hospital, Shanghai Jiao Tong University School of Medicine, Shanghai, China

**Keywords:** Parkinson’s disease, REM sleep behavior disorder (RBD), cognition, nomogram, mild cognitive impairment

## Abstract

The presentation and progression of Parkinson’s disease (PD) are not uniform, but the presence of rapid eye movement sleep behavior disorder (RBD) in PD patients may indicate a worse prognosis than isolated PD. Increasing evidence suggests that patients with comorbid PD and RBD (PD-RBD) are more likely to develop cognitive impairment (CI) than those with isolated PD; however, the predictors of CI in PD-RBD patients are not well understood. This study aimed to develop a prognostic model for predicting mild cognitive impairment (MCI) in PD-RBD patients. The data of PD-RBD patients were extracted from the Parkinson’s Progression Markers Initiative study (PPMI), and the sample was randomly divided into a training set (*n* = 96) and a validation set (*n* = 24). PD-MCI as defined by the level II Movement Disorder Society (MDS) diagnostic criteria was the outcome of interest. The demographic features, clinical assessments, dopamine transporter (DAT) imaging data, cerebrospinal fluid (CSF) analyses and genetic data of PD patients were considered candidate predictors. We found that performance on the University of Pennsylvania Smell Identification Test (UPSIT), the mean signal and asymmetry index of the putamen on DAT imaging, p-tau/α-syn and p-tau in CSF, and rs55785911 genotype were predictors of PD-MCI in PD-RBD patients. A C-index of 0.81 was obtained with this model, and a C-index of 0.73 was obtained in the validation set. Favorable results of calibrations and decision curve analysis demonstrated the efficacy and feasibility of this model. In conclusion, we developed a prognostic model for predicting MCI in PD-RBD patients; the model displayed good discrimination and calibration and may be a convenient tool for clinical application. Larger samples and external validation sets are needed to validate this model.

## Introduction

Parkinson’s disease (PD) is the second most common neurodegenerative disease affecting elderly people worldwide and is mainly characterized by motor symptoms, including bradykinesia, resting tremor, rigidity, and postural instability. Moreover, PD is considered to be a complex and multifaceted disorder with many nonmotor symptoms (NMSs), such as constipation, cognitive impairment (CI), anxiety, and rapid eye movement (REM) sleep behavior disorder (RBD; Kalia and Lang, 2015). CI is an important and common NMS of PD, and studies have shown that the prevalence rate of CI in PD patients rises as the disease progresses, reaching 80% at 20 years since diagnosis (Aarsland et al., 2003; Hely et al., 2008). PD-CI is classified into mild CI in PD (PD-MCI) and PD with dementia (PDD; Litvan et al., 2012); PD-MCI represents the earlier of the two stages and is a risk factor for PDD (Janvin et al., 2006). The presence of CI in PD patients severely aggravates the burden on the patients and their families; therefore, early detection of PD-MCI, even before the transition stage of PDD, is necessary and vital for potential disease prevention and treatment.

RBD is a parasomnia characterized by a loss of REM sleep-related normal skeletal muscle atonia with remarkable motor activities and dream enactment behaviors (Stefani et al., 2018), and the presence of RBD in individuals indicates an increased risk of developing α-synucleinopathy, including PD (Mahowald and Schenck, 2013). Moreover, RBD is a common symptom in PD patients, and it is estimated that the frequency of RBD is approximately 33–48% in PD patients (De Cock et al., 2007; Sixel-Döring et al., 2011; Jozwiak et al., 2017). Studies have shown that patients with comorbid PD and RBD (PD-RBD) have certain characteristics; they tend to be elderly males with non-tremor-dominant or akinetic-rigid motor subtypes and may represent a more severe disease course than isolated PD, correlated with increased autonomic dysfunction, psychiatric comorbidities and falls, along with accelerated cognitive decline and degraded sleep quality (Massicotte-Marquez et al., 2008; Postuma et al., 2008; Kim and Jeon, 2014; Chahine et al., 2016; Pagano et al., 2018; Trout et al., 2019). Therefore, researchers have suggested that PD-RBD may be a relatively diffuse and complex subtype of PD (Postuma et al., 2008, 2012; Lin and Chen, 2018).

Moreover, in terms of cognition, there is a considerable amount of evidence suggesting that PD-RBD is a distinct subtype with worse cognitive outcomes than PD/non-RBD (PD-nRBD; Sinforiani et al., 2006, 2008; Vendette et al., 2007; Gagnon et al., 2009; Postuma et al., 2012; Manni et al., 2013; Nomura et al., 2017), and the presence of RBD in PD is predictive of progression in CI (Sinforiani et al., 2008; Manni et al., 2013; Nomura et al., 2013). PD-RBD patients were reported to perform worse on executive functioning, attention, verbal learning and memory, and visuospatial processing tasks than PD-nRBD patients (Gagnon et al., 2009; Postuma et al., 2012; Jozwiak et al., 2017; Kamble et al., 2019; Yan et al., 2019); however, Postuma et al. (2012) reported that PD-RBD patients showed no difference from PD-nRBD patients in nonverbal learning and memory tasks, indicating a particular CI pattern in PD-RBD patients. In 2012, Postuma et al. (2012) reported that PD-RBD patients with MCI at baseline were more prone to develop dementia than PD-nRBD patients in a 4-year follow-up study. However, to our knowledge, a prospective study investigating whether PD-RBD patients were more likely to develop MCI than PD-nRBD patients over a sufficiently long period has not yet been conducted.

Currently, the factors contributing to the development of CI in PD-RBD patients are not well understood. To our knowledge, no study has investigated risk factors for CI in PD-RBD patients. This study aims to develop and validate a prognostic model with comprehensive assessments for predicting PD-MCI in PD-RBD patients. In addition, the predictors identified in this study could contribute to a better understanding of the PD-RBD phenotype and may guide the prevention of MCI in PD-RBD patients.

## Materials and Methods

### Study Design and Participants

The Parkinson’s Progression Markers Initiative (PPMI) is a multicenter (throughout the United States, Europe, and Australia) longitudinal study with the aim of identifying biomarkers of PD progression (Parkinson Progression Marker Initiative., 2011). Written informed consent was provided by the PPMI participants, and the PPMI study was approved by the institutional boards of the study sites (Weintraub et al., 2015). The detailed methodology and information of the study assessments are available on the PPMI website^1^. The data were updated on April 20th, 2020, and were downloaded from the PPMI website on September 29th, 2020. The participants were enrolled and screened at baseline, which was between June 2011 and April 2013, and were followed up annually.

The REM Sleep Behavior Disorder Questionnaire (RBDSQ) is a widely used and accepted tool to evaluate RBD symptoms and has been validated in multiple populations (Stiasny-Kolster et al., 2007; Nomura et al., 2011; Wang et al., 2015; Ye et al., 2020). In this study, RBDSQ scores ≥5 were defined as probable RBD by PPMI standards, as previously described (Pagano et al., 2018), and PD patients with this condition were defined as PD-RBD patients. For this study, we included PD-RBD patients meeting the following criteria: (1) RBDSQ score ≥5 at baseline and follow-up visits; (2) the status of cognition was recorded in the subsequent visits; and (3) clinical assessment, dopamine transporter (DAT) imaging, cerebrospinal fluid (CSF), and genetic data were available at baseline. Patients with MCI at baseline or without subsequent records of cognition status were excluded from this study. Moreover, to prove that PD-RBD patients had a higher risk of CI, PD-nRBD patients were included. They met the same inclusion and exclusion criteria for this study as the PD-RBD group except for RBDSQ scores <5.

A total of 120 PD-RBD patients and 218 PD-nRBD patients with available baseline clinical assessment, DAT scans, CSF analyses, and genetic data were included in this study. The patients were followed up, and their cognitive status was evaluated at 1-year intervals after baseline enrollment.

### Outcome

The outcome was defined as PD-MCI. In this study, PD-MCI was defined by the PPMI protocol and the Movement Disorder Society (MDS) MCI task force level II guidelines and was originally recorded in the PPMI dataset (Litvan et al., 2011). The cognitive domains in the MDS MCI task force level II guidelines were assessed as follows in PPMI data: the Hopkins Verbal Learning Test (HVLT) total recall and HVLT recognition discrimination were used for verbal memory, Benton Judgment of Line Orientation (JOLO) was used for visuospatial processing, Letter-Number Sequencing (LNS) was used for executive function and working memory, the Semantic fluency Test (SFT) was used for speeded lexical search, and the Symbol-Digit Modalities Test (SDMT) was used for processing speed and attention (Parkinson Progression Marker Initiative., 2011; Schrag et al., 2017; Hogue et al., 2018).

According to the PPMI reference manual, PD-MCI was defined as scores on two or more of HVLT immediate/total recall, HVLT recognition discrimination, JOLO, LNS, SFT, and SDMT that were more than 1.5 standard deviations below normal, with no functional impairment due to CI. These criteria have been applied and validated in several studies (Parkinson Progression Marker Initiative., 2011; Schrag et al., 2017; Hogue et al., 2018).

### Candidate Predictors

The assessments of participants in the PPMI baseline data, including demographic features, clinical assessment, DAT-SCAN, CSF, and genetic data, were considered candidate predictors. The demographic variables were age, gender, and years of education (age and years of education were transformed to categorized variables in PPMI at given cutoff values).

The clinical assessment variables were disease duration, age at symptom onset, and initial symptoms at diagnosis. PD symptoms were assessed using Hoehn and Yahr staging, Total Rigidity Scores, the tremor dominant/postural instability and gait difficulty (TD/PIGD) classification, tremor scores, and Movement Disorder Society Unified Parkinson’s Disease Rating Scale (MDS-UPDRS) scores. The modified Schwab and England Activities of Daily Living (ADL) score was used to evaluate performance in ADL. Olfactory function was assessed with the University of Pennsylvania Smell Identification Test (UPSIT). Depression was assessed with the 15-item Geriatric Depression Scale (GDS). Sleep disturbance was assessed with the Epworth Sleepiness Scale (ESS). Autonomic function was assessed with the Scale for Outcomes in PD for Autonomic Symptoms (SCOPA-AUT). The anxiety level was assessed with the State-Trait Anxiety Inventory (STAI).

For DAT scan imaging data, we included the mean caudate and putaminal uptake relative to uptake in the occipital area and asymmetry of caudate and putaminal uptake. For CSF data, Aβ, α-synuclein, tau, and phosphorylated tau (p-tau) and their relative ratios were included. For genetic data, APOE 4 status, MAPT genotype, and SNPs recorded in PPMI were included (Nalls et al., 2016).

### Statistical Analysis

The original categorical or ranked variables in the PPMI data were retained, while other continuous numeric variables were transformed into ordinal categorical variables according to best cutoffs produced by X-tile software for a better calibration ability of the model (Camp et al., 2004). The endpoint was defined as the time from baseline normal cognition to the occurrence of PD-MCI. We compared MCI-free survival between the PD-RBD group and the PD-nRBD group by Kaplan–Meier and log-rank tests.

We used two methods for predictor selection. Univariate Cox proportional hazards regression analysis was repeated to identify variables associated with MCI-free survival, and variables with significant *P* values (*P* < 0.01) were considered for entry into the multivariate model. The least absolute shrinkage and selection operator (LASSO) regression model was also used to select optimal candidate variables from the high-dimensional data, and variables with nonzero coefficients in LASSO regression were selected for further analysis (Friedman et al., 2010). The final model was formulated on the basis of the results of multivariate Cox regression by using backward stepwise elimination with Akaike information criteria (AIC) as the stopping rule (Ji et al., 2019). The predictive ability of the two models was assessed with the receiver operating characteristic curve (ROC) at 3, 5, and 7 years (Chen et al., 2019).

The best prediction model was visualized with a nomogram for predicting 3-, 5- and, 7-year MCI-free survival probabilities (Iasonos et al., 2008; Balachandran et al., 2015). The concordance index (C-index) was used to evaluate the discrimination of the models (Pencina and D’Agostino, 2004), and calibration curves were plotted to evaluate the calibration of the model (Kramer and Zimmerman, 2007). Decision curves were plotted to assess the clinical usefulness of the model in different years (Vickers and Elkin, 2006).

Statistical analysis was performed using R software (Version 3.6.1). *P* < 0.05 was considered statistically significant.

## Results

### Participants Characteristics

**Figure 1:**
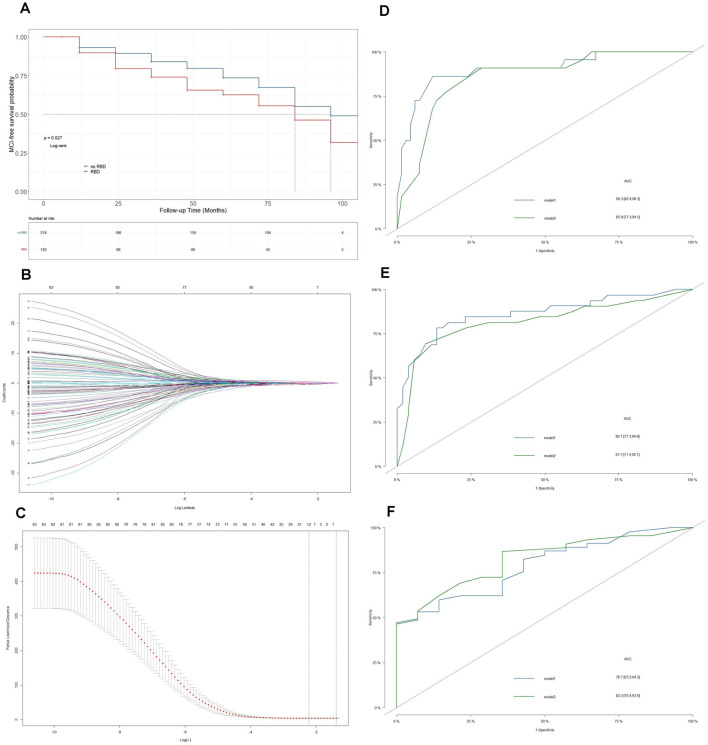
Establishment of the prediction model. **(A)** Kaplan–Meier plots of mild cognitive impairment (MCI)-free survival between Parkinson’s Disease (PD)-RBD patients and PD-nRBD patients. **(B)** Optimal parameter selection in the least absolute shrinkage and selection operator (LASSO) regression model. **(C)** The coefficient profiles of 83 variables in the LASSO model. The lambda we selected resulted in six variables with nonzero coefficients. **(D)** ROC curves of model 1 and model 2 for predicting MCI in PD-RBD patients at 3 years. **(E)** ROC curves of model 1 and model 2 for predicting MCI in PD-RBD patients at 5 years. **(F)** ROC curves of model 1 and model 2 for predicting MCI in PD-RBD patients at 7 years.

**Figure 2:**
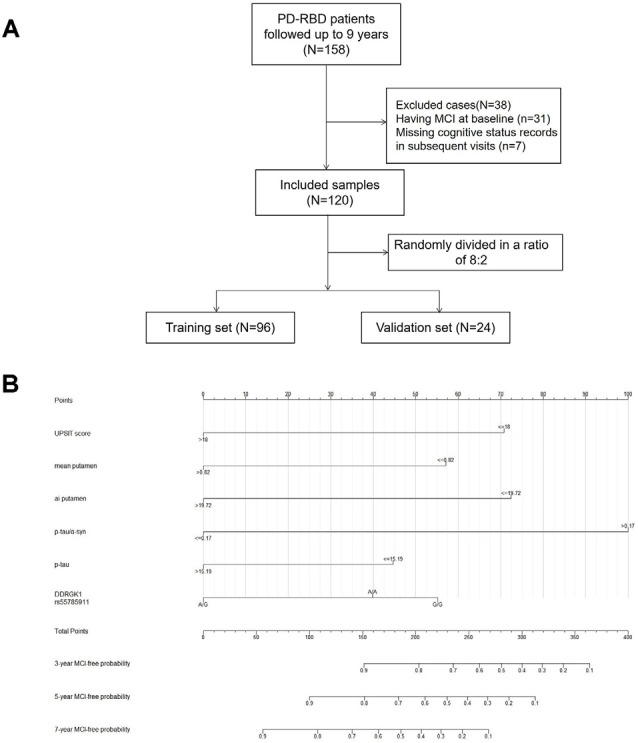
Flow diagram of patient selection and allocation **(A)** and nomogram for predicting the MCI-free probabilities at 3, 5, and 7 years in PD-RBD patients **(B)**. Each level of every predictor corresponds to a score on the points scale, and the total points were acquired by adding the scores of each variable. The total points for each patient correspond to the estimation of the 3-, 5-, and 7-year probabilities of MCI-free survival in this nomogram. Abbreviations: PD, Parkinson’s disease; RBD, Rapid eye movement sleep behavior disorder; MCI, mild cognitive impairment; ai, asymmetry index.

There were 423 PD patients at baseline in the PPMI database. Among the 158 PD-RBD patients, 31 PD-RBD patients were diagnosed with MCI at baseline, seven patients had no cognitive status records at the subsequent visits, and they were removed from further analysis. The sample finally included 120 PD-RBD patients and 218 PD-nRBD patients. For model development and internal validation, we randomly divided the sample into a training set (*n* = 96) and a validation set (*n* = 24) at a ratio of 8:2 (Figure 2A). Kaplan–Meier analysis indicated median MCI-free survival times of 6 years in the PD-RBD group and 7 years in the PD-nRBD group (*P* = 0.029, Figure 1A), suggesting a worse prognosis in the PD-RBD group than in the PD-nRBD group. Cox regression showed that RBD was a predictor of MCI after adjustment for age, sex, and education (HR = 1.43, 95% CI: *P* = 0.029).

The patients were mainly male (65%) and over 65 years old, and most of them had received over 13 years of education. Most of their ages at symptom onset were over 55 years old, and their Hoehn and Yahr stages were all at or below level II (Table 1). Forty-nine patients in the training set and 13 patients in the validation set developed MCI.

**Table 1:** Basic characteristics of PD-RBD patients with different cognitive outcomes.

	Patients developed MCI (*n* = 62)	Patients did not develop MCI (*n* = 58)	*P* value
Age (years)		
<55	20 (34.5%)	15 (24.2%)
55–65	18 (31%)	18 (29%)	0.327
>65	20 (34.5%)	29 (46.8%)
Gender			
Male	32 (55.2%)	46 (74.2%)	0.046
Female	26 (44.8%)	16 (25.8%)
Education level		
Primary or secondary (<13 years of schooling)	9 (15.5%)	6 (9.7%)
Tertiary (13–23 years of schooling)	49 (85.5%)	56 (90.3%)	0.49
Age at symptom onset (years)		
<55	24 (41.4%)	17 (27.4%)	0.156
>55	34 (58.6%)	45 (72.6%)
Hoehn and Yahr stage		
I	32 (55.2%)	26 (41.9%)	0.205
II	26 (44.8%)	36 (58.1%)

*Data are presented as n (%) and compared using the chi-square test*.

### Development of the Prediction Model

The predictors selected from univariate Cox regression and multivariate Cox regression models (model 1) are presented in Table 2. UPSIT score (HR = 0.36, *P* = 0.001), mean signal (HR = 0.44, *P* = 0.001), and asymmetry index of the putamen (HR = 0.35, *P* < 0.001) on DAT imaging, CSF p-tau/α-syn (HR = 4.27, *P* = 0.001), and p-tau (HR = 0.52, *P* = 0.05), and DDRGK1 rs55785911 genotype (HR_AG_ = 0.45, *P* = 0.016; HR_AA_ = 0.89, *P* = 0.66) were found to be final predictors of MCI in PD-RBD patients. A lambda value of 0.15 was applied to select candidate predictors (Figures 1B,C). The final predictors selected from the LASSO regression and multivariate Cox regression models (model 2) were hyposmia (HR = 0.55, *P* = 0.45) and anosmia (HR = 1.28, *P* = 0.75) on the UPSIT, mean signal (HR = 0.4, *P* = 0.007) and asymmetry index of the putamen (HR = 0.37, *P* = 0.001) on DAT imaging, and CSF p-tau (HR = 3.74, *P* = 0.001).

**Table 2:** Univariate and multivariate Cox regression analysis based on predictors of MCI in model 1.

Characteristic	Univariate analysis			Multivariate analysis		
	Coefficient	HR (95%CI)	*P* value	Coefficient	HR (95% CI)	*P* value
UPSIT score						0.001
≤18		Reference			Reference
>18	−0.69	0.5 (0.3, 0.83)	0.007	−1.03	0.36 (0.19, 0.66)
Mean putamen DAT signal						0.001
≤0.82		Reference			Reference
>0.82	−1.05	0.35 (0.2, 0.64)	0.001	−0.82	0.44 (0.23, 0.85)
Asymmetry index of putamen DAT signal						0.001
≤19.72		Reference			Reference
>19.72	−0.82	0.44 (0.26, 0.73)	0.002	−1.05	0.35 (0.19, 0.66)
p-tau/α-syn						0.001
≤0.17		Reference			Reference
>0.17	1.08	2.94 (1.7, 5.06)	0.001	1.45	4.27 (2.2, 8.27)
p-tau						0.05
≤15.19		Reference			Reference
>15.19	−0.45	0.64 (0.39, 1.06)	0.081	−0.65	0.52 (0.27, 1.02)
DDRGK1 rs55785911					
G/G		Reference			Reference
A/G	−0.89	0.41 (0.24, 0.7)	0.001	−0.8	0.45 (0.23, 0.86)	0.016
A/A	−0.2	0.82 (0.38, 1.79)	0.623	−0.22	0.8 (0.29, 2.2)	0.665

ROC curves were plotted to compare the predictive abilities of the two models (Figures 1D–F). Model 1 exhibited a higher area under the curve (AUC) than model 2 at 3 years (90.3 vs. 85.9) and 5 years (86.1 vs. 81.7). Model 1 also had a lower AIC than model 2 (350 vs. 357).

### Nomogram Construction

Model 1 was visualized with a nomogram (Figure 2B). Each level of every predictor corresponds to a score on the points scale, and the total points were acquired by adding the scores of each variable. The total points for each patient correspond to the estimation of the 3-year, 5-year, and 7-year MCI-free probabilities.

For example, suppose a PD-RBD patient scored 15 on the UPSIT, with a mean putamen DAT signal of 1, a putamen asymmetry index of 20 on DAT imaging, a p-tau/α-syn value of 0.5 and a p-tau level of 10 pg/ml in CSF, and had the G/G genotype of DDRGK1 rs55785911. This patient would receive a total score of 270 (UPSIT: 71; mean putamen DAT signal: 0; asymmetry index of putamen: 0; p-tau/α-syn: 100; p-tau: 44; DDRGK1 rs55785911: 55), and the corresponding MCI-free survival probabilities at 3 years and 5 years would be approximately 55% and 28%, respectively.

### Discrimination, Calibration, Clinical Usefulness, and Validation

**Figure 3:**
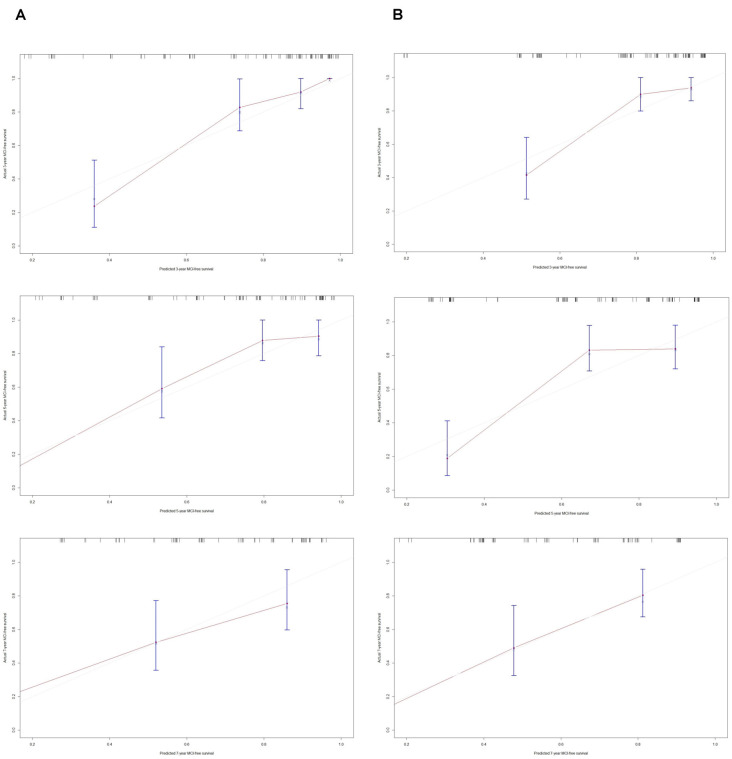
Calibration plots of model-predicted MCI-free survival probabilities at 3 years, 5 years, and 7 years in PD-RBD patients. Model-predicted probabilities of overall survival are plotted on the x-axis; actual overall survival is plotted on the y-axis. **(A)** The calibration curve of model 1-predicted MCI-free survival probabilities at 3 years, 5 years, and 7 years in the training set, *n* = 96. **(B)** The calibration curve of the model 2-predicted probability of MCI-free survival probabilities at 3 years, 5 years, and 7 years in the training set, *n* = 96.

**Figure 4:**
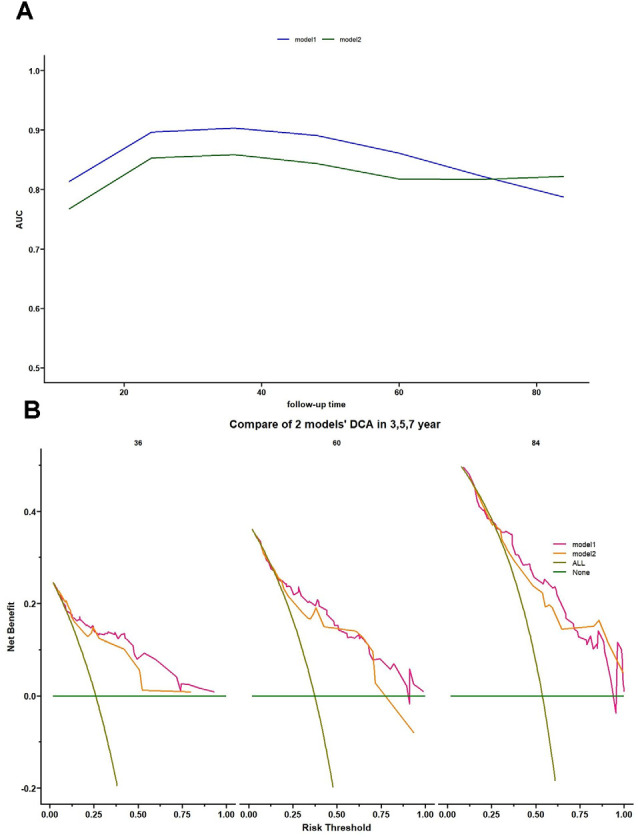
Time-dependent AUC and decision curve analysis (DCA) for model 1 and model 2. **(A)** Time-dependent AUC plot showing how the AUCs of model 1 and model 2 changed with follow-up time. **(B)** DCA of model 1 and model 2 for 3-, 5-, and 7-year predictions. The brown line represents the assumption that all PD-RBD patients have MCI. DCA showed the following: (1) all patients would benefit from model 1 and model 2 for 3-year prediction; (2) for 5-year prediction, patients at risk threshold <0.75 would benefit from model 2, while patients at almost all risk thresholds would gain a positive net benefit in model 1; and (3) using model 1 and model 2 benefits patients more than the treat-all scheme or the treat-none scheme at almost all risk thresholds.

The C-index of model 1 was 0.811 (95% CI: 0.797–0.824, *P* < 0.001), and the C-index of model 2 was 0.782 (95% CI: 0.768–0.796, *P* < 0.01). The calibration plots of model 1 (Figure 3A) and model 2 (Figure 3B) at 3 years, 5 years, and 7 years suggested a better calibration of model 1. AUCs at different follow-up times were plotted for both models, and it was shown that model 1 always had AUCs higher than 0.75 (Figure 4A), and the AUC of model 1 was mostly higher than that of model 2. DCA at 3 years and 7 years showed that high proportions of patients would benefit from the two models. However, the net benefit is above zero only if the risk threshold of model 2 is <0.75 at 5 years. In general, the clinical usefulness of model 1 was better than that of model 2 (Figure 4B).

**Figure 5:**
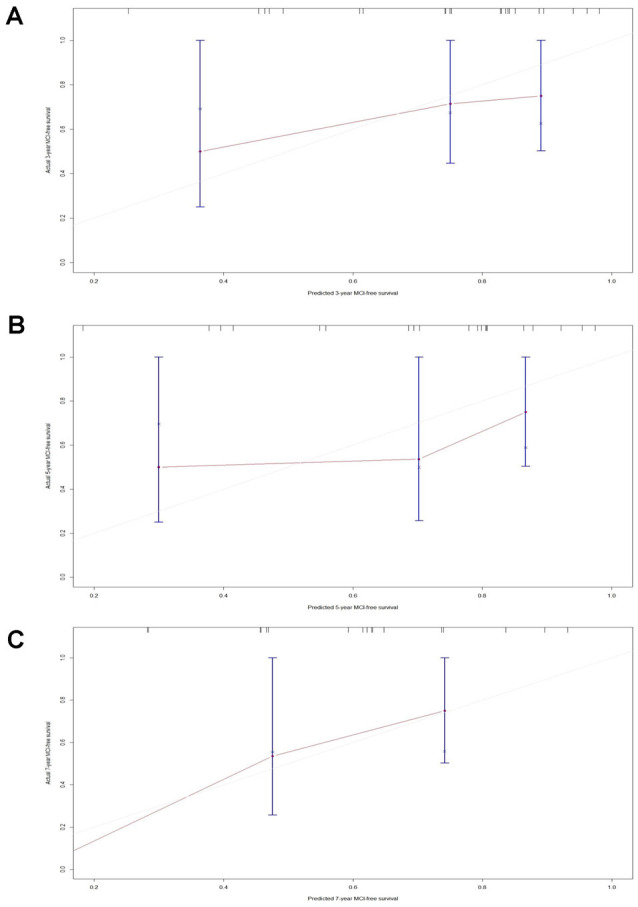
Calibration plots of model 1-predicted probabilities of MCI-free survival at 3 years, 5 years, and 7 years in the validation set. Model-predicted probabilities of overall survival are plotted on the x-axis; actual overall survival is plotted on the y-axis. **(A)** The calibration curve of the model 1-predicted probability of MCI-free survival at 3 years in the validation set, *n* = 24. **(B)** The calibration curve of the model 1-predicted probability of MCI-free survival at 5 years in the validation set, *n* = 24. **(C)** The calibration curve of the model 1-predicted probability of MCI-free survival at 7 years in the validation set, *n* = 24.

A C-index of 0.725 (95% CI: 0.65–0.80, *P* = 0.017) could still be obtained in the validation set. Moreover, the calibration plots of the validation set showed favorable calibration of model 1 at 3, 5, and 7 years (Figure 5).

## Discussion

MCI is common in PD patients, with a prevalence rate of 18.9–38.2%, and is known to be a risk factor for PDD (Litvan et al., 2011). Early diagnosis of MCI is important for clinicians to provide prompt intervention, and a means of estimating the probability of developing MCI helps both patients and clinicians. PD-RBD was reported to be a diffuse and malignant subtype of PD with more rapid progression in cognition and other NMSs (Postuma et al., 2009; Fereshtehnejad et al., 2015, 2017). PD-RBD patients had an increased risk of developing MCI, as confirmed in several studies (Massicotte-Marquez et al., 2008; Postuma et al., 2012; Manni et al., 2013; Jozwiak et al., 2017), and the coexistence of PD and RBD increased the risk of PDD (Anang et al., 2014). Chahine et al. (2016) first investigated the characteristics of PD-RBD patients from the PPMI database, and they found that PD-RBD patients had a greater rate of decline in global cognition than PD-nRBD patients from the 3-year follow-up data. Their conclusions were similar to those of this study, while we evaluated the patients *via* 9-year follow-up data. This study found that PD-RBD patients had a poorer prognosis of cognition in the 9-year cohort of PPMI. A prediction model conducted by Hogue O et al. showed that RBD was not significantly associated with early cognitive decline (Hogue et al., 2018). The sample they used was also from PPMI *de nova* PD patients, and their definition of early cognitive decline was the same as that in this study. However, the different evaluation times may lead to differences in our results. The prediction model developed by Schrag A showed that the RBDSQ score was associated with CI with an odds ratio of 1.13 at 2 years (Schrag et al., 2017), and a meta-analysis showed that the relative ratio of RBD for PD-CI was between 1.2–11.54, which supported our results (Manni et al., 2013; Guo et al., 2020).

In this study, we used two methods for candidate predictor selection, developed two models, and compared their performance. Model 1, which is based on univariate Cox regression, performed better than model 2 in terms of calibration, discrimination, and clinical usefulness. The C-index calculated from the training set and validation set suggested the favorable predictive ability of this model, and the calibration plots of both the training set and validation set were favorable. Although the AUC of model 1 decreased slightly with follow-up time, the overall AUC was >0.75. Moreover, the clinical usefulness suggested by DCA ensured that a large proportion of patients benefited from this model at subsequent visits.

UPSIT performance was found to be a predictor of PD-MCI in this study, and this result is corroborated by several other studies (Hu et al., 2014; Fullard et al., 2016; Hogue et al., 2018; Roos et al., 2019; Pekel et al., 2020). Moreover, olfactory deficits were found to be a risk and predictor of dementia and PD in iRBD patients (Postuma et al., 2019; Lyu et al., 2021). It has been reported that PD-MCI is more likely to be associated with severe olfactory impairment than PD patients with normal cognition (Park et al., 2018), and an imaging study found entorhinal cortex atrophy in early, drug-naive PD patients with MCI (Jia et al., 2019). Moreover, a study suggested that cortical thinning in the olfactory cortex is a marker for early dementia conversion in PD-MCI (Chung et al., 2019). The mechanisms behind this are still unclear, but deterioration in olfactory function has been suggested to reflect extrastriatal neurodegeneration in PD, and olfactory impairment suggests early involvement of the olfactory bulb in PD (Fullard et al., 2017).

Patients in the early stages of PD often show decreased levels of DAT uptake, especially in the putamen (Benamer et al., 2000). A cohort study followed for 5.5 years found that lower striatal binding at baseline was associated with a higher risk for CI in de novo PD patients (Ravina et al., 2012). Similarly, this study found that a lower mean signal and asymmetry index of the putamen on DAT imaging were correlated with a higher risk of developing MCI. Moreover, it has been reported that PD-RBD patients exhibit a more rapid decrease in DAT binding, and the distinct pattern of striatal DAT binding may contribute to the more malignant subtype—PD-RBD (Cao et al., 2020).

P-tau was reported to induce defective autophagy and mitophagy in Alzheimer’s disease (Reddy and Oliver, 2019) and was considered a potential biomarker for PD (Parnetti et al., 2019). A meta-analysis revealed that higher CSF p-tau could be observed in a PDD cohort than in PD patients with normal cognition, while CSF levels of p-tau between PD-CI patients and PD patients with normal cognition were not statistically significant (Hu et al., 2017). In this study, a higher ratio of p-tau/α-synuclein was found to be a risk factor for PD-MCI, and this may be specific to this PD-RBD subtype. The role of p-tau in this nomogram was merely to improve the performance of the model, as it was not statistically significant. Future studies with other cohorts are needed to validate the role of CSF p-tau/α-synuclein.

rs55785911/DDRGK1 was an SNP recorded in PPMI SNP data. A large-scale meta-analysis of genome-wide association data has identified DDRGK1 as a risk locus for PD (Nalls et al., 2014; Wang et al., 2016). DDRGK1 is an endoplasmic reticulum membrane protein and a critical component of the ubiquitin-fold modifier 1 (Ufm1) system and is critical for endoplasmic reticulum homeostasis (Liu et al., 2017). Ming Zou et al. investigated the association of variants of SIPA1 L2, MIR4697, GCH1, VPS13C, and DDRGK1 with PD in East Asians; however, they found that rs8118008/DDRGK1 was not correlated with PD (Wang et al., 2016). However, in this study, rs55785911/DDRGK1 was found to contribute to this model, and interestingly, patients with the A/G genotype had a better cognitive prognosis than patients with the G/G genotype. Although no studies have investigated the role of rs55785911/DDRGK1 in PD, the results need to be further confirmed because the results may be caused by sample size, and considering that overall 50 SNPs were tested, it is possible that some of the results were due to chance. Future studies with larger cohorts are needed.

There are several limitations to this study. First, seven patients were excluded from this study for missing cognitive status records in subsequent visits, which may cause bias to this study. Second, this model was only validated internally and should be validated in a different cohort. Third, the patients in this study were de novo and newly diagnosed patients without formal polysomnography (PSG) proven (which is absent from the PPMI database), but many studies using PPMI data were the same as this study, we all used RBDSQ score ≥5 as the cutoff value, and this criterion was validated in several populations. Therefore, this study pioneered further PSG-proven studies. Finally, the sample size of this study was limited.

The aim of this study was to develop a prognostic model for PD-RBD patients to predict the risk of MCI, and as a result, the model showed favorable discrimination, calibration, and clinical usefulness even at 7 years. In conclusion, this study is the first to develop and validate a prognostic model for de novo PD-RBD patients to practically assess MCI-free survival probabilities. This study demonstrated risk factors for MCI in the PD-RBD subgroup, thus contributing insights into the PD-RBD phenotype. As new disease-modifying treatments are being developed, strategies to identify PD-RBD patients at high risk of developing MCI could be useful to prevent or decelerate the progression of MCI in PD-RBD patients. Future studies are needed to validate the model and explore the role of predictors in this model.

## Data Availability Statement

Publicly available datasets were analyzed in this study. This data can be found here: http://ppmi-info.org.

## Ethics Statement

The data included in this study are from the Parkinson’s Progression Markers Initiative (PPMI) database. For the ethics statements, please see the detailed in http://www.ppmi-info.org.

## Author Contributions

FC: executed statistical analysis and wrote the first draft. YL: reviewed and critiqued the statistical analysis and the manuscript. GY and LZ: reviewed, critiqued, and revised the manuscript. JL and XB: produced the concept of this project, reviewed, and critiqued the manuscript. All authors contributed to the article and approved the submitted version.

## Conflict of Interest

The authors declare that the research was conducted in the absence of any commercial or financial relationships that could be construed as a potential conflict of interest.

## Footnotes

^1^http://ppmi-info.org
